# The Effects of Previous Thyroid Disease on the Susceptibility to, Morbidity of, and Mortality Due to COVID-19: A Nationwide Cohort Study in South Korea

**DOI:** 10.3390/jcm10163522

**Published:** 2021-08-11

**Authors:** So-Young Kim, Dae-Myoung Yoo, Chan-Yang Min, Hyo-Geun Choi

**Affiliations:** 1 CHA Bundang Medical Center, Department of Otorhinolaryngology-Head & Neck Surgery, CHA University, Seongnam 13496, Korea; sossi81@hanmail.net; 2 Hallym Data Science Laboratory, Hallym University College of Medicine, Anyang 14068, Korea; ydm1285@naver.com (D.-M.Y.); joicemin@naver.com (C.-Y.M.); 3 Graduate School of Public Health, Seoul National University, Seoul 08826, Korea; 4 Department of Otorhinolaryngology-Head & Neck Surgery, Hallym University College of Medicine, Anyang 14068, Korea

**Keywords:** thyroid, morbidity, mortality, COVID-19, case-control studies, cohort studies

## Abstract

This study aimed to investigate the associations of the susceptibility to, morbidity of, and mortality due to coronavirus disease 2019 (COVID-19) with thyroid diseases. Korea National Health Insurance Database Coronavirus disease 2019 (NHID-COVID-19) medical claim code data from 2015 to 2020 were analyzed. A total of 8070 COVID-19 patients and 32,280 matched control participants were evaluated for histories of hypothyroidism, hyperthyroidism, Graves’ disease, thyroiditis, and autoimmune thyroiditis. The relationships of susceptibility to, morbidity of, and mortality due to COVID-19 with hypothyroidism, hyperthyroidism, Graves’ disease, thyroiditis, and autoimmune thyroiditis were analyzed using a conditional logistic regression. Hypothyroidism, hyperthyroidism, Graves’ disease, thyroiditis, and autoimmune thyroiditis were not associated with susceptibility to, morbidity of, or mortality due to COVID-19. Graves’ disease was related to higher odds of mortality due to COVID-19 in the adjusted model but the confidence interval (CI) was wide, probably due to the small number of deaths among patients with Graves’ disease (aOR = 11.43, 95% CI = 1.29–101.22, *p* = 0.029). Previous histories of hypothyroidism, hyperthyroidism, Graves’ disease, thyroiditis, and autoimmune thyroiditis were not related to susceptibility to COVID-19. In addition, prior histories of thyroid diseases were not related to increased risks of COVID-19-related morbidity and mortality.

## 1. Introduction

Coronavirus disease 2019 (COVID-19) is a respiratory infectious disease caused by severe acute respiratory syndrome coronavirus 2 (SARS-CoV-2) [1]. Although the primary route of SARS-CoV-2 entry is the respiratory system, viral infection is known to affect multiple organs and systems including the cardiovascular, respiratory, neurological, and gastrointestinal systems [2,3,4]. In addition, the functioning of multiple organ systems is required to fight SARS-CoV-2 infection [2]. Therefore, the dysfunction of these organs could impair recovery from COVID-19. Indeed, many previous studies have reported an increased risk of COVID-19 in patients with chronic diseases such as diabetes, obesity, coronary artery disease, and hypertension [5].

The risk of COVID-19 in patients with thyroid diseases has been suggested [6,7]. First, angiotensin-converting enzyme 2 (ACE2) receptors are known to play a role in the entry of SARS-CoV-2 [8]. Thyroid parenchyma have abundant ACE2 receptors [6]. Thus, the role of the thyroid in the modulation of ACE2 levels and the entry of SARS-CoV-2 has been proposed [6]. Second, abnormalities in the thyroid function have been acknowledged to impact multiple organ systems such as the cardiovascular and respiratory systems [9,10]. Third, approximately 95% of hypothyroidism cases and 50% of hyperthyroidism cases are attributed to autoimmunity [11,12]. Although the risk of SARS-CoV-2 infection in patients with autoimmune diseases is still unclear, a weakened immune system due to immune suppressant use could result in an increased morbidity of COVID-19 [13]. For these reasons, it can be supposed that an impaired thyroid function may exacerbate SARS-CoV-2 infection [14]. Indeed, a number of papers have described a thyroid dysfunction following SARS-CoV-2 infection [15,16]. Multiple case series have reported the subsequent relapse of Graves’ disease following SARS-CoV-2 infection [13,14].

The impacts of preexisting thyroid diseases on susceptibility to SARS-CoV-2 and morbidity of COVID-19 are controversial [17,18]. Uncontrolled thyrotoxicosis, Graves’ ophthalmopathy, and neutropenia induced by antithyroid drugs have been suggested to increase the risk of COVID-19 morbidity whereas autoimmune thyroid disease and hyperthyroidism have not been shown to increase the risk of COVID-19 morbidity [17]. Therefore, different associations of each type of thyroid disease with COVID-19 could be expected.

We hypothesized that the impact of thyroid diseases on COVID-19 could vary according to the type of thyroid disease. The primary objective of this study was to evaluate the association of previous thyroid diseases with susceptibility to COVID-19 in all participants. The secondary objective was to estimate the relationship between previous thyroid diseases and morbidity/mortality in COVID-19 patients.

## 2. Materials and Methods

### 2.1. Ethics

The ethics committee of Hallym University (2020-07-022) permitted this study following the guidelines and regulations. Written informed consent was waived by the Institutional Review Board.

### 2.2. Study Population and Participant Selection

We analyzed Korea National Health Insurance Database Coronavirus disease 2019 (NHID-COVID-19) medical claim code data from 2015 to 2020. NHID-COVID-19 contains the data of individuals who underwent SARS-CoV-2 testing using real-time reverse transcriptase (RT)-PCR testing of nasal or pharyngeal swabs in accordance with the World Health Organization (WHO) guideline; the data of control participants were proportionally sampled from the database of the Korean National Health Insurance and were stratified by age and sex.

**Figure 1:**
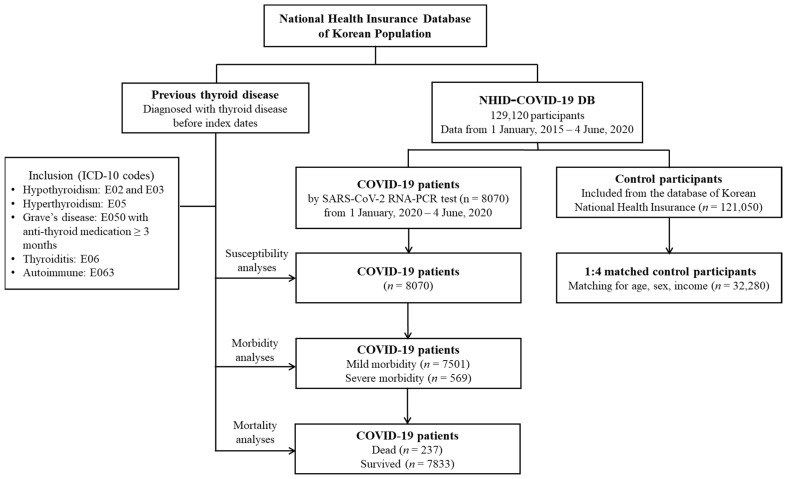
A schematic illustration of the participant selection process that was used in the present study. Of a total of 129,120 participants, 8070 COVID-19 patients were matched with 32,280 control participants for age, sex, and income. ICD-10: International Classification of Diseases, revision 10.

Confirmed COVID-19 patients from 1 January 2020 to 4 June 2020 were included and all of them finished treatment or died by 4 June 2020 (*n* = 8070). The number of control participants initially selected was fifteen-fold higher than that of the confirmed COVID-19 patients (*n* = 121,050). We matched COVID-19 patients with control participants in a 1:4 ratio in terms of age, sex, and income. Among the control participants, we excluded participants with a lack of income records (*n* = 2136). To avoid selection bias, control participants were selected randomly using clustered sampling. The index date was defined as the date of confirmation of COVID-19. The index dates for the control participants were randomly selected and ranged from 1 January 2020 to 4 June 2020. Finally, 8070 COVID-19 participants were 1:4 matched with 32,280 control participants. The prevalence rates of previous thyroid diseases were analyzed in the COVID-19 and control participants. The COVID-19 patients were then assessed to determine mild (*n* = 7501) and severe (*n* = 569) morbidity. The rates of death (*n* = 237) and survival (*n* = 7833) in COVID-19 patients were also analyzed (Figure 1).

### 2.3. Exposure (Thyroid Diseases)

In our study, hypothyroidism, hyperthyroidism, Graves’ disease, thyroiditis, and autoimmune thyroiditis were included in the studied thyroid diseases.

1. Hypothyroidism was defined as International Classification of Diseases 10th revision (ICD-10) codes E02 (subclinical iodine-deficiency hypothyroidism) and E03 (other hypothyroidism). Among the participants with these codes, we selected the participants who were treated ≥ 2 times.
2. Hyperthyroidism was defined as ICD-10 code E05 (hyperthyroidism (thyrotoxicosis)). Among these participants, we selected the participants who were treated ≥ 2 times.
3. Graves’ disease was defined as ICD-10 code E050 (thyrotoxicosis with diffuse goiter). Among these participants, we selected those who were treated with antithyroid medication ≥ 3 months.
4. Thyroiditis was defined as ICD-10 code E06 (thyroiditis). Among these participants, we selected the participants who were treated ≥ 2 times.
5. Autoimmune thyroiditis was defined as ICD-10 code E063 (autoimmune thyroiditis). Among these participants, we selected the participants who were treated ≥ 2 times.

### 2.4. Outcome (COVID-19 Infection)

Laboratory confirmation of SARS-CoV-2 infection was defined as the primary outcome.

### 2.5. Secondary Outcomes (Morbidity and Mortality)

The secondary outcomes were morbidity and mortality among COVID-19 patients. Morbidity was defined as either severe or mild morbidity. Severe morbidity was defined as admission to an intensive care unit (ICU), invasive ventilation, extracorporeal membrane oxygenation (ECOM), or death.

### 2.6. Covariates

Age groups were divided into 10-year intervals: 0–9, 10–19, 20–29 …, and 80 + years old (total of 9 age groups). Income groups were classified into 3 classes (low income, middle income, and high income). Those with missing income (*n* = 127 (0.31%)) data were included in the middle income group. The Charlson comorbidity index (CCI) was considered a continuous variable (0 (no comorbidities) to 29 (multiple comorbidities)) [19]. Regarding thyroid diseases, hypertension (ICD-10 codes I10 and I15) was additionally assigned if participants were treated ≥ 2 times.

### 2.7. Statistical Analyses

The general characteristics between the COVID-19 groups and control groups and between the severe morbidity groups and mild morbidity groups were compared using the chi-squared test or Fisher’s exact test.

To estimate susceptibility in COVID-19 patients compared to control participants, odds ratios (ORs) with 95% confidence intervals (CIs) for thyroid diseases were calculated using a crude (simple) model, model 1 (adjusted for CCI score and hypertension), model 2 (adjusted for the variables in model 1 plus hypothyroidism, hyperthyroidism, and thyroiditis), and model 3 (adjusted for the variables in model 1 plus hypothyroidism, Graves’ disease, and autoimmune thyroiditis) with a conditional logistic regression. In these analyses, age, sex, and income were stratified. To estimate morbidity/mortality in the COVID-19 patients according to the type of thyroid disease, an unconditional logistic regression was used in the nonmatched analyses. For the subgroup analyses, we stratified participants by age (<50 years old and ≥50 years old), sex, income (low, middle, and high), CCI scores (0 score, 1 score, and ≥2 score), and hypertension history.

For the statistical analyses, SAS version 9.4 (SAS Institute Inc., Cary, NC, USA) was used. We performed two-tailed analyses and significance was defined as a *p*-value less than 0.05.

## 3. Results

Histories of hypothyroidism and hyperthyroidism were more common in the COVID-19 group than in the control group (20.5% vs. 20.0%, *p* = 0.026 for hypothyroidism and 1.7% vs. 1.6%, *p* = 0.010 for hyperthyroidism; Table 1). The COVID-19 group had a higher CCI score than the control group (*p* < 0.001).

Hypothyroidism was associated with higher odds of COVID-19 infection in the crude model (OR = 1.17, 95% CI = 1.02–1.34, *p* = 0.025; Table 2). However, the relationship of hypothyroidism with COVID-19 infection was not maintained in model 1, model 2, and model 3. The other types of thyroid diseases, such as hyperthyroidism, Graves’ disease, thyroiditis, and autoimmune thyroiditis, were not associated with COVID-19 infection. There were no relationships between thyroid diseases and COVID-19 infection in all age, sex, income, CCI score, and hypertension subgroups (Supplementary Materials Table S1).

A total of 7.05% (569/8070) of COVID-19 patients had severe morbidity (Table 1). There was no significant difference in the rate of hypothyroidism, hyperthyroidism, Graves’ disease, thyroiditis, or autoimmune thyroiditis between the severe and mild COVID-19 groups. The severe COVID-19 group had a higher rate of hypertension and a higher CCI score than the mild COVID-19 group (both *p* < 0.001). Hypothyroidism, hyperthyroidism, Graves’ disease, thyroiditis, and autoimmune thyroiditis were not associated with severe COVID-19 morbidity in the crude model, model 1, model 2, and model 3 (Table 3). The subgroup with a CCI score = 0 had increased odds for severe COVID-19 morbidity related to hypothyroidism (adjusted OR (aOR) = 1.79, 95% CI = 1.02–3.15, *p* = 0.044 in model 3; Supplementary Materials Table S2). The other (age, sex, income, CCI score, and hypertension) subgroups did not show relationships between thyroid diseases and COVID-19 infection.

Graves’ disease was associated with increased odds of mortality due to COVID-19, but the CI was wide (aOR = 11.43, 95% CI = 1.29–101.22, *p* = 0.029; Table 4). Mortality due to COVID-19 was not associated with hyperthyroidism or hypothyroidism. The relationships of thyroiditis and autoimmune thyroiditis with mortality due to COVID-19 could not be evaluated due to a lack of fatal cases in each thyroid disease group.

## 4. Discussion

Hypothyroidism, hyperthyroidism, Graves’ disease, thyroiditis, and autoimmune thyroiditis were not associated with COVID-19 infection in the Korean population. In addition, morbidity of and mortality due to COVID-19 were not related to these types of thyroid diseases. This study expands the knowledge gained from previous studies as it analyzed the associations of susceptibility to, morbidity of, and mortality due to COVID-19 with several types of thyroid diseases.

Several epidemiological studies have reported nonsignificant associations of preexisting thyroid diseases with COVID-19 similar to the present results [20,21,22]. In a case-control study in Europe, patients with hypothyroidism or hyperthyroidism did not show an increased risk of SARS-CoV-2 infection and treatment for a thyroid dysfunction did not decrease the risk of COVID-19 morbidity [20]. In a retrospective study in the US, patients with hypothyroidism did not have higher risks of hospitalization, mechanical ventilation, or mortality due to COVID-19 [21]. A retrospective study in Iran reported that COVID-19 patients with preexisting hypothyroidism did not have higher rates of morbidity and mortality than other COVID-19 patients [22]. There may be mixed impacts of thyroid diseases on the risk of COVID-19 due to multiple pathophysiologic factors.

Several pathophysiologic mechanisms may increase the risk of COVID-19 in patients with thyroid diseases. ACE2 expression, which is known to facilitate SARS-CoV-2 entry, has been shown to be high in patients with a thyroid dysfunction [23]. In addition, hyperthyroidism-induced hypercoagulable states increase the levels of coagulation factors such as factor (F) VIII, FX, FIX, von Willebrand F, and fibrinogen [24]. COVID-19 also induces hypercoagulability, potentially resulting in microangiopathy and thrombus formation [25]. Furthermore, the thyroid has a protective role in the lung function [26,27]. In animal studies, a thyroid dysfunction impaired fluid clearance and induced lung fibrosis [26,27]. Thus, high levels of ACE2, augmented hypercoagulability, and compromised protective effects of the lungs in patients with a thyroid dysfunction could increase the rates of morbidity and mortality associated with COVID-19.

On the other hand, a few factors alleviate the risk of COVID-19 in patients with thyroid diseases. Another protein that is required for internalization of SARS-CoV-2, integrin αvβ3, is a competitive inhibitor of L-thyroxine (T4) [28]. By competitively binding with integrin αvβ3, T4 regulates the internalization of SARS-CoV-2 and modulates the affinity of integrin with other proteins, which can induce cytokine storms in COVID-19 patients. Thus, it has been suggested that thyroid hormone analogs can inhibit SARS-CoV-2 infection and morbidity of or mortality due to COVID-19. In addition, positive health-seeking behavior and precautions against SARS-CoV-2 infection in patients with thyroid diseases could attenuate the risk of SARS-CoV-2 infection. Patients with chronic diseases are presumed to have a higher risk of SARS-CoV-2 infection than those without chronic diseases and patients with thyroid diseases may have more opportunities to obtain knowledge about SARS-CoV-2 preventive behaviors from their healthcare providers [29,30]. Moreover, medications for underlying thyroid diseases could mitigate the potential risk for COVID-19 by maintaining euthyroid states. Last, the proportions of COVID-19-related morbidity and mortality were not high enough in our cohort to demonstrate statistically significant differences in COVID-19-related morbidity or mortality in patients with thyroid diseases.

This study analyzed data from a large, nationwide cohort population. The control group was matched by demographic characteristics with the COVID-19 group. In addition, the relationships of several types of thyroid diseases with susceptibility to, morbidity of, and mortality due to COVID-19 were explored. However, some information was deficient in this study due to the nature of medical health claim data. The results of thyroid function tests and medication histories, such as thyroid hormone replacement therapy, were unavailable. Subclinical or undiagnosed cases of thyroid disease could not be accounted for. Although the relationships of preexisting thyroid diseases with the occurrence of SARS-CoV-2 infection and morbidity of and mortality due to COVID-19 were analyzed, the duration from the thyroid disease onset to the SARS-CoV-2 infection onset was heterogeneous; therefore, causality between thyroid disease and SARS-CoV-2 infection could not be delineated. Long-term follow-up data will be helpful in elucidating the impacts of thyroid diseases on the disease course of COVID-19.

## 5. Conclusions

Preexisting hypothyroidism, hyperthyroidism, Graves’ disease, thyroiditis, and autoimmune thyroiditis were not associated with the susceptibility to, morbidity of, or mortality due to COVID-19 in the Korean population. Patients with thyroid diseases could be managed for COVID-19 without concern for the additional risks of COVID-19 compared to the general population.

## Figures and Tables

**Table 1:** General Characteristics of Participants.

Characteristics		Total Participants			COVID-19 Participants		
		COVID-19	Control	*p*-Value	Severe Morbidity	Mild Morbidity	*p*-Value
Total number (*n*, %)		8070 (100.0)	32,280 (100.0)		569 (100.0)	7501 (100.0)	
Age (years old) (*n*, %)				1.000			<0.001 *
	0–9	81 (1.0)	324 (1.0)		6 (1.1)	75 (1.0)	
	10–19	276 (3.4)	1104 (3.4)		6 (1.1)	270 (3.6)	
	20–29	2057 (25.5)	8228 (25.5)		31 (5.5)	2026 (27.0)	
	30–39	832 (10.3)	3328 (10.3)		25 (4.4)	807 (10.8)	
	40–49	1036 (12.8)	4144 (12.8)		30 (5.3)	1006 (13.4)	
	50–59	1567 (19.4)	6268 (19.4)		71 (12.5)	1496 (19.9)	
	60–69	1199 (14.9)	4796 (14.9)		116 (20.4)	1083 (14.4)	
	70–79	617 (7.7)	2468 (7.7)		118 (20.7)	499 (6.7)	
	80 +	405 (5.0)	1620 (5.0)		166 (29.2)	239 (3.2)	
Sex (*n*, %)				1.000			<0.001 *
	Male	3236 (40.1)	12,944 (40.1)		306 (53.8)	2930 (39.1)	
	Female	4834 (59.9)	19,336 (59.9)		263 (46.2)	4571 (60.9)	
Income (*n*, %)				1.000			0.029 *
	1 (low)	3105 (38.5)	12,420 (38.5)		196 (34.5)	2909 (38.8)	
	2	2347 (29.1)	9388 (29.1)		161 (28.3)	2186 (29.1)	
	3 (high)	2618 (32.4)	10,472 (32.4)		212 (37.3)	2406 (32.1)	
CCI score (*n*, %)				<0.001 *			<0.001 *
	0	6518 (80.8)	29,513 (91.4)		264 (46.4)	6254 (83.4)	
	1	889 (11.0)	1389 (4.3)		134 (23.6)	755 (10.1)	
	≥ 2	663 (8.2)	1378 (4.3)		171 (30.1)	492 (6.6)	
Hypertension (*n*, %)		1657 (20.5)	6452 (20.0)	0.274	275 (48.3)	1382 (18.4)	<0.001 *
Hypothyroidism (*n*, %)		284 (3.5)	980 (3.0)	0.026 *	23 (4.0)	261 (3.5)	0.483
Hyperthyroidism (*n*, %)		134 (1.7)	531 (1.6)	0.010 *	10 (1.8)	124 (1.7)	0.851
Graves’ disease (*n*, %)		20 (0.3)	97 (0.3)	0.431	1 (0.2)	19 (0.3)	0.720
Thyroiditis (*n*, %)		108 (1.3)	423 (1.3)	0.844	5 (0.9)	103 (1.4)	0.322
Autoimmune thyroiditis (*n*, %)		52 (0.6)	155 (0.5)	0.065	3 (0.5)	49 (0.7)	0.717

CCI: Charlson comorbidity index; COVID-19: coronavirus disease 2019. * Chi-squared or Fisher’s exact test. Significance at *p* < 0.05.

**Table 2:** Odds ratios of each thyroid disease for COVID-19 infection in all participants.

Characteristics	COVID-19	Control	ORs (95% Confidence Interval) for COVID-19							
	(Exposure/Total, %)	(Exposure/Total, %)	Crude †	*p*-Value	Model 1 †,‡	*p*-Value	Model 2 †,§	*p*-Value	Model 3 †,‖	*p*-Value
Hypothyroidism	284/8070 (3.5%)	980/32,280 (3.0%)	1.17 (1.02–1.34)	0.025 *	1.11 (0.97–1.28)	0.126	1.12 (0.97–1.29)	0.120	1.09 (0.95–1.26)	0.229
Hyperthyroidism	134/8070 (1.7%)	531/32,280 (1.6%)	1.01 (0.83–1.22)	0.922	1.00 (0.82–1.21)	0.983	0.98 (0.81–1.20)	0.871	N/A	
Graves’ disease	20/8070 (0.2%)	97/32,280 (0.3%)	0.82 (0.51–1.34)	0.432	0.82 (0.51–1.33)	0.423	N/A		0.79 (0.49–1.29)	0.348
Thyroiditis	108/8070 (1.3%)	423/32,280 (1.3%)	1.02 (0.83–1.27)	0.844	1.01 (0.81–1.25)	0.930	0.98 (0.78–1.23)	0.840	N/A	
Autoimmune thyroiditis	52/8070 (0.6%)	155/32,280 (0.5%)	1.35 (0.98–1.85)	0.065	1.32 (0.96–1.82)	0.085	N/A		1.27 (0.92–1.77)	0.146

CCI: Charlson comorbidity index; N/A: not applicable. * Conditional logistic regression, significance at *p* < 0.05. † Models were stratified by age, sex, and income. ‡ Model 1 was adjusted for CCI scores and hypertension. § Model 2 was adjusted for model 1 plus hypothyroidism, hyperthyroidism, and thyroiditis. ‖ Model 3 was adjusted for model 1 plus hypothyroidism, Graves’ disease, and autoimmune thyroiditis.

**Table 3:** Odds ratios of each thyroid disease for morbidity in COVID-19 participants.

Characteristics	Severe Participants	Mild Participants	ORs (95% Confidence Interval) for Morbidity							
	(Exposure/Total, %)	(Exposure/Total, %)	Crude	*p*-Value	Model 1 †	*p*-Value	Model 2 ‡	*p*-Value	Model 3 §	*p*-Value
Hypothyroidism	23/569 (4.0%)	261/7501 (3.5%)	1.17 (0.76–1.81)	0.483	0.98 (0.62–1.57)	0.946	1.01 (0.62–1.63)	0.978	0.99 (0.61–1.61)	0.977
Hyperthyroidism	10/569 (1.8%)	124/7501 (1.7%)	1.06 (0.56–2.04)	0.851	1.19 (0.60–2.35)	0.616	1.25 (0.63–2.50)	0.526	N/A	
Graves’ disease	1/569 (0.2%)	19/7501 (0.3%)	0.69 (0.09–5.19)	0.722	0.90 (0.12–6.98)	0.919	N/A		0.93 (0.12–7.49)	0.946
Thyroiditis	5/569 (0.9%)	103/7501 (1.4%)	0.64 (0.26–1.57)	0.327	0.73 (0.29–1.86)	0.511	0.69 (0.26–1.82)	0.457	N/A	
Autoimmune thyroiditis	3/569 (0.5%)	49/7501 (0.7%)	0.81 (0.25–2.59)	0.718	0.89 (0.27–2.96)	0.850	N/A		0.90 (0.26–3.15)	0.870

CCI: Charlson comorbidity index; N/A: not applicable. † Model 1 was adjusted for age, sex, income, CCI scores, and hypertension. ‡ Model 2 was adjusted for model 1 plus hypothyroidism, hyperthyroidism, and thyroiditis. § Model 3 was adjusted for model 1 plus hypothyroidism, Graves’ disease, and autoimmune thyroiditis.

**Table 4:** Odds ratios of each thyroid disease for mortality in COVID-19 participants.

Characteristics	Dead Participants	Survived Participants	ORs (95% Confidence Interval) for Mortality							
	(Exposure/Total, %)	(Exposure/Total, %)	Crude	*p*-Value	Model 1 †	*p*-Value	Model 2 ‡	*p*-Value	Model 3 §	*p*-Value
Hypothyroidism	6/237 (2.5%)	278/7833 (3.5%)	0.71 (0.31–1.60)	0.406	0.48 (0.19–1.20)	0.115	0.51 (0.20–1.28)	0.152	0.51 (0.20–1.27)	0.147
Hyperthyroidism	4/237 (1.7%)	130/7833 (1.7%)	1.02 (0.37–2.78)	0.973	1.69 (0.55–5.17)	0.359	2.32 (0.74–7.30)	0.152	N/A	
Graves’ disease	1/237 (0.4%)	19/7833 (0.2%)	1.74 (0.23–13.07)	0.589	5.77 (0.70–47.74)	0.104	N/A		11.43 (1.29–101.22)	0.029 *
Thyroiditis	0/237 (0.0%)	108/7833 (1.4%)	N/A		N/A		N/A		N/A	
Autoimmune thyroiditis	0/237 (0.0%)	52/7833 (0.7%)	N/A		N/A		N/A		N/A	

CCI: Charlson comorbidity index; N/A: not applicable. * Unconditional logistic regression model, significance at *p* < 0.05. † Model 1 was adjusted for age, sex, income, CCI scores, and hypertension. ‡ Model 2 was adjusted for model 1 plus hypothyroidism, hyperthyroidism, and thyroiditis. § Model 3 was adjusted for model 1 plus hypothyroidism, Graves’ disease, and autoimmune thyroiditis.

## Data Availability

Releasing of the data by the researcher is not legally permitted. All data are available from the database of the Korea Center for Disease Control and Prevention. The Korea Center for Disease Control and Prevention allows data access, at a particular cost, for any researcher who promises to follow the research ethics. The data of this article can be downloaded from the website after agreeing to follow the research ethics.

## Supplementary Materials

The following are available online at https://www.mdpi.com/article/10.3390/jcm10163522/s1. Table S1: Stratified subgroup analyses of odd ratios of each thyroid disease for COVID-19 infection in the total participants by covariates; Table S2: Stratified subgroup analyses of odd ratios of each thyroid disease for morbidity in COVID-19 participants by covariates; Table S3: Stratified subgroup analyses of odd ratios of each thyroid disease for mortality in COVID-19 participants by covariates.
