# Supplementary material for: The Effects of Previous Thyroid Disease on the Susceptibility to, Morbidity of, and Mortality Due to COVID-19: A Nationwide Cohort Study in South Korea
Source: J Clin Med. 2021 Aug 11;10(16):3522. doi: 10.3390/jcm10163522 (PMC8396860; doi:10.3390/jcm10163522)
Supplement: Supplementary file 1 [file jcm-10-03522-s001.zip › jcm-1336125-supplementary.pdf]

Table S1. Stratified subgroup analyses of odd ratios of each thyroid disease for COVID-19 infection in total participants in total participants by covariates

Characteristics	COVID-19 (exposure/total, %)	Control (exposure/total, %)	ORs (95% confidence interval) for COVID-19							
			Crude†	P-value	Model 1†‡	P-value	Model 2†§	P-value	Model 3†‖	P-value
**Age < 50 years old (n = 21,410)**										
Hypothyroidism	97/4,282 (2.3%)	319/17,128 (1.9%)	0.82 (0.65-1.03)	0.085	0.88 (0.69-1.11)	0.266	1.17 (0.92-1.50)	0.203	1.16 (0.91-1.48)	0.243
Hyperthyroidism	49/4,282 (1.1%)	218/17,128 (1.3%)	1.12 (0.82-1.52)	0.497	1.16 (0.85-1.59)	0.348	0.85 (0.61-1.18)	0.328	N/A	
Grave's disease	9/4,282 (0.2%)	52/17,128 (0.3%)	1.45 (0.71-2.94)	0.307	1.49 (0.73-3.03)	0.274	N/A		0.65 (0.32-1.33)	0.241
Thyroiditis	43/4,282 (1.0%)	176/17,128 (1.0%)	1.02 (0.73-1.43)	0.891	1.04 (0.74-1.46)	0.807	0.95 (0.66-1.37)	0.798	N/A	
Autoimmune thyroiditis	19/4,282 (0.4%)	68/17,128 (0.4%)	0.89 (0.54-1.49)	0.667	0.95 (0.57-1.58)	0.833	N/A		1.00 (0.58-1.69)	0.985
**Age ≥ 50 years old (n = 18,940)**										
Hypothyroidism	187/3,788 (4.9%)	661/15,152 (4.4%)	0.88 (0.74-1.04)	0.125	0.91 (0.77-1.08)	0.287	1.09 (0.92-1.30)	0.332	1.06 (0.89-1.26)	0.511
Hyperthyroidism	85/3,788 (2.2%)	313/15,152 (2.1%)	0.92 (0.72-1.17)	0.493	0.91 (0.72-1.17)	0.473	1.08 (0.84-1.39)	0.563	N/A	
Grave's disease	11/3,788 (0.3%)	45/15,152 (0.3%)	1.02 (0.53-1.98)	0.947	1.01 (0.52-1.97)	0.971	N/A		0.95 (0.49-1.84)	0.869
Thyroiditis	65/3,788 (1.7%)	247/15,152 (1.6%)	0.95 (0.72-1.25)	0.710	0.96 (0.72-1.26)	0.753	1.00 (0.75-1.34)	0.996	N/A	
Autoimmune thyroiditis	33/3,788 (0.9%)	87/15,152 (0.6%)	0.66 (0.44-0.98)	0.041*	0.65 (0.43-0.98)	0.038*	N/A		1.49 (0.98-2.26)	0.059
**Men (n = 16,180)**										
Hypothyroidism	44/3,236 (1.4%)	142/12,944 (1.1%)	0.80 (0.57-1.13)	0.209	0.85 (0.60-1.21)	0.366	1.20 (0.84-1.71)	0.321	1.08 (0.75-1.55)	0.695
Hyperthyroidism	18/3,236 (0.6%)	89/12,944 (0.7%)	1.24 (0.75-2.06)	0.410	1.25 (0.75-2.08)	0.403	0.79 (0.47-1.34)	0.386	N/A	
Grave's disease	2/3,236 (0.1%)	18/12,944 (0.1%)	2.25 (0.52-9.71)	0.276	2.38 (0.55-10.30)	0.247	N/A		0.42 (0.10-1.81)	0.242
Thyroiditis	14/3,236 (0.4%)	57/12,944 (0.4%)	1.02 (0.57-1.83)	0.953	1.04 (0.58-1.88)	0.894	0.95 (0.51-1.75)	0.866	N/A	
Autoimmune thyroiditis	10/3,236 (0.3%)	14/12,944 (0.1%)	0.35 (0.16-0.79)	0.011*	0.37 (0.16-0.83)	0.016*	N/A		2.61 (1.11-6.14)	0.028*
**Women (n = 24,170)**										
Hypothyroidism	240/4,834 (5.0%)	838/19,336 (4.3%)	0.87 (0.75-	0.056	0.91 (0.78-1.05)	0.194	1.11 (0.95-	0.202	1.09 (0.94-1.28)	0.257

			1.00)				1.29)			
Hyperthyroidism	116/4,834 (2.4%)	442/19,336 (2.3%)	0.95 (0.77-1.17)	0.637	0.96 (0.78-1.19)	0.727	1.02 (0.82-1.27)	0.831	N/A	
Grave's disease	18/4,834 (0.4%)	79/19,336 (0.4%)	1.10 (0.66-1.83)	0.722	1.09 (0.65-1.83)	0.739	N/A		0.89 (0.53-1.50)	0.669
Thyroiditis	94/4,834 (1.9%)	366/19,336 (1.9%)	0.97 (0.77-1.22)	0.814	0.98 (0.78-1.24)	0.879	0.98 (0.77-1.25)	0.858	N/A	
Autoimmune thyroiditis	42/4,834 (0.9%)	141/19,336 (0.7%)	0.84 (0.59-1.19)	0.317	0.85 (0.60-1.20)	0.355	N/A		1.13 (0.79-1.62)	0.500
**Low income (n = 15,525)**										
Hypothyroidism	122/3,105 (3.9%)	368/12,420 (3.0%)	0.74 (0.60-0.92)	0.006*	0.79 (0.63-0.97)	0.028	1.29 (1.03-1.60)	0.024*	1.26 (1.01-1.57)	0.038*
Hyperthyroidism	54/3,105 (1.7%)	215/12,420 (1.7%)	1.00 (0.74-1.35)	0.975	1.02 (0.75-1.39)	0.886	0.95 (0.69-1.30)	0.745	N/A	
Grave's disease	5/3,105 (0.2%)	33/12,420 (0.3%)	1.65 (0.64-4.24)	0.296	1.68 (0.65-4.33)	0.281	N/A		0.57 (0.22-1.47)	0.243
Thyroiditis	40/3,105 (1.3%)	160/12,420 (1.3%)	1.00 (0.71-1.42)	1.000	0.99 (0.70-1.41)	0.971	0.94 (0.65-1.36)	0.753	N/A	
Autoimmune thyroiditis	19/3,105 (0.6%)	59/12,420 (0.5%)	0.78 (0.46-1.30)	0.335	0.77 (0.46-1.30)	0.334	N/A		1.16 (0.68-1.98)	0.592
**Middle income (n = 11,735)**										
Hypothyroidism	81/2,347 (3.5%)	288/9,388 (3.1%)	0.88 (0.69-1.14)	0.337	0.94 (0.72-1.21)	0.608	1.06 (0.82-1.38)	0.656	1.03 (0.79-1.34)	0.843
Hyperthyroidism	40/2,347 (1.7%)	150/9,388 (1.6%)	0.94 (0.66-1.33)	0.714	0.93 (0.65-1.33)	0.688	1.06 (0.73-1.54)	0.753	N/A	
Grave's disease	10/2,347 (0.4%)	34/9,388 (0.4%)	0.85 (0.42-1.72)	0.651	0.85 (0.42-1.74)	0.663	N/A		1.13 (0.55-2.30)	0.747
Thyroiditis	35/2,347 (1.5%)	131/9,388 (1.4%)	0.93 (0.64-1.36)	0.724	0.96 (0.66-1.40)	0.831	1.00 (0.67-1.50)	0.989	N/A	
Autoimmune thyroiditis	18/2,347 (0.8%)	48/9,388 (0.5%)	0.67 (0.39-1.15)	0.141	0.69 (0.40-1.20)	0.185	N/A		1.42 (0.80-2.50)	0.228
**High income (n = 13,090)**										
Hypothyroidism	81/2,618 (3.1%)	324/10,472 (3.1%)	1.00 (0.78-1.28)	1.000	1.02 (0.75-1.39)	0.886	0.98 (0.75-1.26)	0.850	0.95 (0.73-1.24)	0.711
Hyperthyroidism	40/2,618 (1.5%)	166/10,472 (1.6%)	1.04 (0.73-1.47)	0.832	1.05 (0.74-1.49)	0.805	0.96 (0.67-1.38)	0.830	N/A	
Grave's disease	5/2,618 (0.2%)	30/10,472 (0.3%)	1.50 (0.58-3.88)	0.400	1.50 (0.58-3.87)	0.407	N/A		0.66 (0.25-1.70)	0.386
Thyroiditis	33/2,618 (1.3%)	132/10,472 (1.3%)	1.00 (0.68-	1.000	1.02 (0.69-1.51)	0.912	1.00 (0.66-	0.989	N/A	

			1.47)				1.50)			
Autoimmune thyroiditis	15/2,618 (0.6%)	48/10,472 (0.5%)	0.80 (0.45-1.43)	0.449	0.81 (0.45-1.46)	0.487	N/A		1.29 (0.70-2.35)	0.412
**CCI scores = 0 (n = 36,031)**										
Hypothyroidism	204/6,233 (3.3%)	821/29,207 (2.8%)	0.89 (0.76-1.04)	0.126	0.84 (0.72-0.98)	0.030*	1.20 (1.02-1.41)	0.031*	1.17 (0.99-1.37)	0.061
Hyperthyroidism	103/6,233 (1.7%)	474/29,207 (1.6%)	1.02 (0.82-1.26)	0.881	0.98 (0.79-1.22)	0.870	0.99 (0.79-1.24)	0.936	N/A	
Grave’s disease	16/6,233 (0.3%)	89/29,207 (0.3%)	1.23 (0.72-2.09)	0.448	1.24 (0.73-2.11)	0.433	N/A		0.78 (0.46-1.33)	0.363
Thyroiditis	85/6,233 (1.4%)	378/29,207 (1.3%)	0.98 (0.78-1.24)	0.879	0.96 (0.76-1.22)	0.744	0.98 (0.76-1.26)	0.886	N/A	
Autoimmune thyroiditis	40/6,233 (0.6%)	138/29,207 (0.5%)	0.76 (0.53-1.08)	0.129	0.74 (0.52-1.06)	0.100	N/A		1.25 (0.87-1.80)	0.237
**CCI scores = 1 (n = 2,278)**										
Hypothyroidism	42/1,110 (3.8%)	75/1,673 (4.5%)	1.15 (0.78-1.70)	0.477	1.08 (0.73-1.60)	0.680	0.89 (0.60-1.33)	0.570	0.87 (0.58-1.30)	0.492
Hyperthyroidism	17/1,110 (1.5%)	24/1,673 (1.4%)	0.90 (0.48-1.69)	0.744	0.89 (0.48-1.68)	0.722	1.06 (0.55-2.05)	0.856	N/A	
Grave’s disease	2/1,110 (0.2%)	4/1,673 (0.2%)	1.28 (0.23-7.01)	0.775	1.29 (0.24-7.05)	0.771	N/A		0.66 (0.12-3.82)	0.647
Thyroiditis	15/1,110 (1.4%)	17/1,673 (1.0%)	0.72 (0.36-1.45)	0.361	0.74 (0.36-1.49)	0.391	1.39 (0.66-2.90)	0.386	N/A	
Autoimmune thyroiditis	07/1,110 (0.6%)	04/1,673 (0.2%)	0.36 (0.11-1.25)	0.108	0.38 (0.11-1.30)	0.124	N/A		2.99 (0.84-10.70)	0.092
**CCI scores ≥ 2 (n = 2,041)**										
Hypothyroidism	38/727 (5.2%)	84/1,400 (6.0%)	1.07 (0.72-1.58)	0.748	1.02 (0.69-1.53)	0.907	1.03 (0.68-1.55)	0.902	0.99 (0.66-1.49)	0.950
Hyperthyroidism	14/727 (1.9%)	33/1,400 (2.4%)	1.14 (0.60-2.14)	0.690	1.04 (0.55-1.97)	0.909	1.09 (0.56-2.12)	0.812	N/A	
Grave’s disease	2/727 (0.3%)	4/1,400 (0.3%)	0.96 (0.18-5.27)	0.964	0.81 (0.15-4.48)	0.813	N/A		1.30 (0.23-7.39)	0.768
Thyroiditis	08/727 (1.1%)	28/1,400 (2.0%)	1.70 (0.77-3.74)	0.190	1.62 (0.73-3.58)	0.235	0.60 (0.26-1.38)	0.225	N/A	
Autoimmune thyroiditis	05/727 (0.7%)	13/1,400 (0.9%)	1.25 (0.45-3.53)	0.669	1.16 (0.41-3.29)	0.778	N/A		0.84 (0.29-2.48)	0.757
**Non-hypertension (n = 32,241)**										
Hypothyroidism	183/6,413 (2.9%)	691/25,824 (2.7%)	0.94 (0.79-1.10)	0.432	0.97 (0.82-1.15)	0.710	1.05 (0.88-1.25)	0.574	1.03 (0.87-1.22)	0.746

Hyperthyroidism	96/6,413 (1.5%)	398/25,824 (1.5%)	1.03 (0.82-1.29)	0.799	1.04 (0.83-1.30)	0.732	0.98 (0.77-1.24)	0.843	N/A	
Grave’s disease	15/6,413 (0.2%)	83/25,824 (0.3%)	1.38 (0.79-2.38)	0.257	1.38 (0.80-2.41)	0.249	N/A		0.71 (0.41-1.24)	0.234
Thyroiditis	76/6,413 (1.2%)	335/25,824 (1.3%)	1.10 (0.85-1.41)	0.475	1.10 (0.85-1.41)	0.464	0.90 (0.69-1.18)	0.448	N/A	
Autoimmune thyroiditis	34/6,413 (0.5%)	125/25,824 (0.5%)	0.91 (0.62-1.33)	0.637	0.93 (0.63-1.36)	0.695	N/A		1.08 (0.73-1.60)	0.694
**Hypertension (n = 8,109)**										
Hypothyroidism	101/1,657 (6.1%)	289/6,456 (4.5%)	0.72 (0.57-0.91)	0.006*	0.75 (0.59-0.95)	0.017*	1.29 (1.01-1.66)	0.040*	1.25 (0.98-1.60)	0.078
Hyperthyroidism	38/1,657 (2.3%)	133/6,456 (2.1%)	0.90 (0.62-1.29)	0.558	0.88 (0.61-1.27)	0.489	1.05 (0.71-1.53)	0.819	N/A	
Grave’s disease	5/1,657 (0.3%)	14/6,456 (0.2%)	0.72 (0.26-2.00)	0.526	0.71 (0.25-1.98)	0.511	N/A		1.30 (0.46-3.64)	0.623
Thyroiditis	32/1,657 (1.9%)	88/6,456 (1.4%)	0.70 (0.47-1.06)	0.089	0.70 (0.46-1.06)	0.093	1.28 (0.82-1.98)	0.276	N/A	
Autoimmune thyroiditis	18/1,657 (1.1%)	30/6,456 (0.5%)	0.43 (0.24-0.77)	0.004*	0.41 (0.23-0.75)	0.003*	N/A		2.12 (1.15-3.92)	0.017*

Abbreviations: CCI, Charlson comorbidity index; N/A, Not applicable

* Conditional logistic regression, Significance at P < 0.05

† Models were stratified by age, sex, and income.

‡ Model 1 was adjusted for CCI scores and hypertension.

§ Model 2 was adjusted for model 1 plus hypothyroidism, hyperthyroidism, and thyroiditis.

‖ Model 3 was adjusted for model 1 plus hypothyroidism, Grave’s disease, and autoimmune thyroiditis.

Table S2. Stratified subgroup analyses of odd ratios of each thyroid disease for morbidity in COVID-19 participants by covariates

Characteristics	Severe participants (exposure/total, %)	Mild participants (exposure/total, %)	ORs (95% confidence interval) for morbidity							
			Crude	P-value	Model 1†	P-value	Model 2‡	P-value	Model 3§	P-value
**Age < 50 years old (n =)**										
Hypothyroidism	3/98 (3.1%)	94/4,184 (2.2%)	0.73 (0.23-2.34)	0.592	0.91 (0.27-3.02)	0.876	0.96 (0.27-3.49)	0.954	1.02 (0.28-3.67)	0.981
Hyperthyroidism	2/98 (2.0%)	47/4,184 (1.1%)	0.55 (0.13-2.28)	0.406	0.72 (0.17-3.12)	0.658	1.19 (0.26-5.55)	0.823	N/A	
Grave's disease	0/98 (0.0%)	9/4,184 (0.2%)	N/A		N/A		N/A		N/A	
Thyroiditis	2/98 (2.0%)	41/4,184 (1.0%)	0.48 (0.11-1.99)	0.308	0.50 (0.12-2.12)	0.350	1.85 (0.37-9.29)	0.453	N/A	
Autoimmune thyroiditis	1/98 (1.0%)	18/4,184 (0.4%)	0.42 (0.06-3.17)	0.400	0.48 (0.06-3.67)	0.481	N/A		2.01 (0.23-17.74)	0.532
**Age ≥ 50 years old (n = 3,788)**										
Hypothyroidism	20/471 (4.2%)	167/3,317 (5.0%)	1.20 (0.74-1.92)	0.461	1.31 (0.80-2.14)	0.288	1.07 (0.63-1.82)	0.812	1.06 (0.62-1.80)	0.838
Hyperthyroidism	8/471 (1.7%)	77/3,317 (2.3%)	1.38 (0.66-2.87)	0.396	1.17 (0.55-2.46)	0.687	1.37 (0.62-3.03)	0.435	N/A	
Grave's disease	1/471 (0.2%)	10/3,317 (0.3%)	1.42 (0.18-11.11)	0.738	1.09 (0.14-8.69)	0.934	N/A		1.93 (0.22-16.97)	0.555
Thyroiditis	3/471 (0.6%)	62/3,317 (1.9%)	2.97 (0.93-9.49)	0.067	2.97 (0.90-9.87)	0.075	0.53 (0.15-1.81)	0.310	N/A	
Autoimmune thyroiditis	2/471 (0.4%)	31/3,317 (0.9%)	2.21 (0.53-9.27)	0.278	2.00 (0.47-8.49)	0.347	N/A		0.69 (0.15-3.27)	0.643
**Men (n = 3,236)**										
Hypothyroidism	8/306 (2.6%)	36/2,930 (1.2%)	0.46 (0.21-1.01)	0.052	0.80 (0.35-1.85)	0.597	1.21 (0.50-2.91)	0.669	1.17 (0.48-2.85)	0.725
Hyperthyroidism	3/306 (1.0%)	15/2,930 (0.5%)	0.52 (0.15-1.81)	0.303	0.62 (0.17-2.29)	0.473	1.62 (0.42-6.33)	0.487	N/A	
Grave's disease	0/306 (0.0%)	2/2,930 (0.1%)	N/A		N/A		N/A		N/A	
Thyroiditis	1/306 (0.3%)	13/2,930 (0.4%)	1.36 (0.18-10.42)	0.768	1.81 (0.23-14.21)	0.574	0.50 (0.06-4.38)	0.535	N/A	
Autoimmune thyroiditis	1/306 (0.3%)	9/2,930 (0.3%)	0.94 (0.12-7.42)	0.952	1.38 (0.17-11.31)	0.763	N/A		0.70 (0.07-6.52)	0.751
**Women (n = 4,834)**										
Hypothyroidism	15/263 (5.7%)	225/4,571 (4.9%)	0.86 (0.50-1.47)	0.571	1.18 (0.67-2.10)	0.570	0.93 (0.52-1.66)	0.802	0.92 (0.51-1.63)	0.763
Hyperthyroidism	7/263 (2.7%)	109/4,571 (2.4%)	0.89 (0.41-1.94)	0.775	0.96 (0.43-2.11)	0.910	1.16 (0.52-2.61)	0.722	N/A	
Grave's disease	1/263 (0.4%)	17/4,571 (0.4%)	0.98 (0.13-7.37)	0.983	0.78 (0.10-6.03)	0.815	N/A		1.20 (0.14-10.11)	0.869
Thyroiditis	4/263 (1.5%)	90/4,571 (2.0%)	1.30 (0.47-3.57)	0.610	1.47 (0.50-4.27)	0.482	0.74 (0.25-2.18)	0.580	N/A	
Autoimmune thyroiditis	2/263 (0.8%)	40/4,571 (0.9%)	1.15 (0.28-4.79)	0.846	1.16 (0.27-4.95)	0.839	N/A		0.94 (0.21-4.32)	0.937
**Low income (n = 3,105)**										
Hypothyroidism	8/196 (4.1%)	114/2,909 (3.9%)	0.96 (0.46-1.99)	0.908	1.53 (0.70-3.36)	0.288	0.71 (0.32-1.61)	0.416	0.72 (0.32-1.63)	0.427
Hyperthyroidism	3/196 (1.5%)	51/2,909 (1.8%)	1.15 (0.36-3.71)	0.818	1.20 (0.36-3.99)	0.761	0.97 (0.29-3.31)	0.964	N/A	
Grave's disease	0/196 (0.0%)	5/2,909 (0.2%)	N/A		N/A		N/A		N/A	

Thyroiditis	2/196 (1.0%)	38/2,909 (1.3%)	1.28 (0.31-5.36)	0.732	1.32 (0.31-5.74)	0.708	1.10 (0.24-5.11)	0.904	N/A	
Autoimmune thyroiditis	1/196 (0.5%)	18/2,909 (0.6%)	1.21 (0.16-9.14)	0.851	1.58 (0.20-12.59)	0.665	N/A		1.07 (0.12-9.46)	0.953
**Middle income (n = 2,347)**										
Hypothyroidism	9/161 (5.6%)	72/2,186 (3.3%)	0.58 (0.28-1.17)	0.128	0.77 (0.36-1.63)	0.496	1.49 (0.67-3.32)	0.330	1.44 (0.63-3.26)	0.388
Hyperthyroidism	3/161 (1.9%)	37/2,186 (1.7%)	0.91 (0.28-2.97)	0.872	1.01 (0.30-3.41)	0.985	0.91 (0.24-3.45)	0.887	N/A	
Grave's disease	0/161 (0.0%)	10/2,186 (0.5%)	N/A		N/A		N/A		N/A	
Thyroiditis	3/161 (1.9%)	32/2,186 (1.5%)	0.78 (0.24-2.58)	0.687	0.76 (0.23-2.54)	0.653	1.53 (0.40-5.83)	0.538	N/A	
Autoimmune thyroiditis	2/161 (1.2%)	16/2,186 (0.7%)	0.59 (0.13-2.57)	0.479	0.63 (0.14-2.80)	0.541	N/A		2.04 (0.38-10.94)	0.405
**High income (n = 2,618)**										
Hypothyroidism	6/212 (2.8%)	75/2,406 (3.1%)	1.11 (0.48-2.57)	0.817	1.35 (0.54-3.37)	0.519	1.08 (0.43-2.72)	0.866	1.00 (0.40-2.48)	0.993
Hyperthyroidism	4/212 (1.9%)	36/2,406 (1.5%)	0.79 (0.28-2.24)	0.657	0.80 (0.27-2.37)	0.680	1.71 (0.57-5.15)	0.342	N/A	
Grave's disease	1/212 (0.5%)	4/2,406 (0.2%)	0.35 (0.04-3.15)	0.350	0.25 (0.03-2.34)	0.222	N/A		10.72 (0.73-156.72)	0.083
Thyroiditis	0/212 (0.0%)	33/2,406 (1.4%)	N/A		N/A		N/A		N/A	
Autoimmune thyroiditis	0/212 (0.0%)	15/2,406 (0.6%)	N/A		N/A		N/A		N/A	
**CCI scores = 0 (n = 6,518)**										
Hypothyroidism	16/240 (6.7%)	188/5,993 (3.1%)	0.48 (0.28-0.81)	0.006*	0.55 (0.32-0.96)	0.034*	1.80 (1.02-3.17)	0.042*	1.79 (1.02-3.15)	0.044*
Hyperthyroidism	8/240 (3.3%)	95/5,993 (1.6%)	0.49 (0.24-1.03)	0.059	0.57 (0.27-1.20)	0.137	1.68 (0.77-3.63)	0.190	N/A	
Grave's disease	1/240 (0.4%)	15/5,993 (0.3%)	0.63 (0.08-4.81)	0.658	0.63 (0.08-5.05)	0.662	N/A		1.48 (0.18-12.14)	0.717
Thyroiditis	4/240 (1.7%)	81/5,993 (1.4%)	0.85 (0.31-2.35)	0.758	0.97 (0.34-2.74)	0.956	0.71 (0.24-2.10)	0.534	N/A	
Autoimmune thyroiditis	3/240 (1.3%)	37/5,993 (0.6%)	0.52 (0.16-1.69)	0.275	0.68 (0.20-2.31)	0.531	N/A		1.05 (0.29-3.79)	0.945
**CCI scores = 1 (n = 889)**										
Hypothyroidism	3/143 (2.1%)	39/967 (4.0%)	2.38 (0.72-7.81)	0.153	1.84 (0.54-6.28)	0.331	0.64 (0.19-2.22)	0.482	0.57 (0.17-1.96)	0.373
Hyperthyroidism	0/143 (0.0%)	17/967 (1.8%)	N/A		N/A		N/A		N/A	
Grave's disease	0/143 (0.0%)	2/967 (0.2%)	N/A		N/A		N/A		N/A	
Thyroiditis	0/143 (0.0%)	15/967 (1.6%)	N/A		N/A		N/A		N/A	
Autoimmune thyroiditis	0/143 (0.0%)	7/967 (0.7%)	N/A		N/A		N/A		N/A	
**CCI scores ≥ 2 (n = 663)**										
Hypothyroidism	4/186 (2.2%)	34/541 (6.3%)	3.10 (1.08-8.86)	0.035	2.62 (0.87-7.88)	0.088	0.37 (0.12-1.16)	0.087	0.42 (0.14-1.27)	0.122
Hyperthyroidism	2/186 (1.1%)	12/541 (2.2%)	2.11 (0.47-9.54)	0.331	1.02 (0.19-5.39)	0.986	1.00 (0.18-5.59)	0.998	N/A	
Grave's disease	0/186 (0.0%)	2/541 (0.4%)	N/A		N/A		N/A		N/A	
Thyroiditis	1/186 (0.5%)	7/541 (1.3%)	2.45 (0.30-20.09)	0.403	1.32 (0.15-11.37)	0.798	1.29 (0.14-12.15)	0.826	N/A	
Autoimmune	0/186 (0.0%)	5/541 (0.9%)	N/A		N/A		N/A		N/A	

thyroiditis										
**Non-hypertension (n = 6,413)**										
Hypothyroidism	11/294 (3.7%)	172/6,119 (2.8%)	0.74 (0.40-1.38)	0.350	0.77 (0.41-1.46)	0.429	1.44 (0.75-2.78)	0.275	1.38 (0.72-2.65)	0.329
Hyperthyroidism	4/294 (1.4%)	92/6,119 (1.5%)	1.11 (0.40-3.03)	0.844	1.23 (0.43-3.47)	0.702	0.87 (0.31-2.50)	0.801	N/A	
Grave's disease	0/294 (0.0%)	15/6,119 (0.2%)	N/A		N/A		N/A		N/A	
Thyroiditis	2/294 (0.7%)	74/6,119 (1.2%)	1.79 (0.44-7.32)	0.419	1.77 (0.43-7.26)	0.431	0.61 (0.14-2.62)	0.505	N/A	
Autoimmune thyroiditis	1/294 (0.3%)	33/6,119 (0.5%)	1.58 (0.22-11.58)	0.651	1.74 (0.23-12.89)	0.589	N/A		0.76 (0.10-5.78)	0.787
**Hypertension (n = 1,657)**										
Hypothyroidism	12/275 (4.4%)	89/1,382 (6.4%)	1.51 (0.81-2.80)	0.192	1.66 (0.87-3.14)	0.122	0.77 (0.39-1.54)	0.462	0.75 (0.38-1.52)	0.428
Hyperthyroidism	6/275 (2.2%)	32/1,382 (2.3%)	1.06 (0.44-2.56)	0.895	0.92 (0.38-2.24)	0.851	1.91 (0.73-4.97)	0.188	N/A	
Grave's disease	1/275 (0.4%)	4/1,382 (0.3%)	0.80 (0.09-7.14)	0.838	0.68 (0.07-6.21)	0.732	N/A		3.06 (0.32-29.53)	0.333
Thyroiditis	3/275 (1.1%)	29/1,382 (2.1%)	1.94 (0.59-6.43)	0.276	1.80 (0.52-6.20)	0.353	0.87 (0.24-3.17)	0.835	N/A	
Autoimmune thyroiditis	2/275 (0.7%)	16/1,382 (1.2%)	1.60 (0.37-6.97)	0.534	1.25 (0.28-5.54)	0.768	N/A		1.26 (0.25-6.33)	0.781

Abbreviations: CCI, Charlson comorbidity index; N/A, Not applicable

* Unconditional logistic regression model, Significance at $P < 0.05$

† Model 1 was adjusted for age, sex, income, CCI scores and hypertension.

‡ Model 2 was adjusted for model 1 plus hypothyroidism, hyperthyroidism, and thyroiditis.

§ Model 3 was adjusted for model 1 plus hypothyroidism, Grave's disease, and autoimmune thyroiditis.

Table S3. Stratified subgroup analyses of odd ratios of each thyroid disease for mortality in COVID-19 participants by covariates

Characteristics	Dead participants	Survived participants	ORs (95% confidence interval) for mortality							
	(exposure/total, %)	(exposure/total, %)	Crude	P-value	Model 1†	P-value	Model 2‡	P-value	Model 3§	P-value
**Age < 50 years old (n =)**										
Hypothyroidism	0/4 (0.0%)	97/4,278 (2.3%)	N/A		N/A		N/A		N/A	
Hyperthyroidism	0/4 (0.0%)	49/4,278 (1.1%)	N/A		N/A		N/A		N/A	
Grave's disease	0/4 (0.0%)	9/4,278 (0.2%)	N/A		N/A		N/A		N/A	
Thyroiditis	0/4 (0.0%)	43/4,278 (1.0%)	N/A		N/A		N/A		N/A	
Autoimmune thyroiditis	0/4 (0.0%)	19/4,278 (0.4%)	N/A		N/A		N/A		N/A	
**Age ≥ 50 years old (n = 3,788)**										
Hypothyroidism	6/233 (2.6%)	181/3,555 (5.1%)	2.03 (0.89-4.63)	0.092	2.51 (1.07-5.93)	0.035	0.52 (0.21-1.30)	0.161	0.51 (0.21-1.29)	0.155
Hyperthyroidism	4/233 (1.7%)	81/3,555 (2.3%)	1.33 (0.49-3.67)	0.577	1.05 (0.37-2.93)	0.930	2.38 (0.75-7.54)	0.141	N/A	
Grave's disease	1/233 (0.4%)	10/3,555 (0.3%)	0.65 (0.08-5.11)	0.684	0.41 (0.05-3.32)	0.404	N/A		15.35 (1.57-150.20)	0.019
Thyroiditis	0/233 (0.0%)	65/3,555 (1.8%)	N/A		N/A		N/A		N/A	
Autoimmune thyroiditis	0/233 (0.0%)	33/3,555 (0.9%)	N/A		N/A		N/A		N/A	
**Men (n = 3,236)**										
Hypothyroidism	4/134 (3.0%)	40/3,102 (1.3%)	0.43 (0.15-1.20)	0.107	0.89 (0.29-2.77)	0.841	1.13 (0.34-3.78)	0.848	1.13 (0.34-3.79)	0.848
Hyperthyroidism	1/134 (0.7%)	17/3,102 (0.5%)	0.73 (0.10-5.54)	0.763	0.96 (0.12-7.86)	0.971	1.13 (0.08-15.24)	0.926	N/A	
Grave's disease	0/134 (0.0%)	2/3,102 (0.1%)	N/A		N/A		N/A		N/A	
Thyroiditis	0/134 (0.0%)	14/3,102 (0.5%)	N/A		N/A		N/A		N/A	
Autoimmune thyroiditis	0/134 (0.0%)	10/3,102 (0.3%)	N/A		N/A		N/A		N/A	
**Women (n = 4,834)**										
Hypothyroidism	2/103 (1.9%)	238/4,731 (5.0%)	2.67 (0.66-10.89)	0.170	5.67 (1.29-24.94)	0.022	0.25 (0.05-1.14)	0.073	0.26 (0.06-1.15)	0.075
Hyperthyroidism	3/103 (2.9%)	113/4,731 (2.4%)	0.82 (0.26-2.61)	0.731	0.83 (0.25-2.74)	0.756	3.31 (0.92-11.96)	0.068	N/A	
Grave's disease	1/103 (1.0%)	17/4,731 (0.4%)	0.37 (0.05-2.79)	0.333	0.20 (0.03-1.66)	0.137	N/A		19.93 (1.80-220.47)	0.015
Thyroiditis	0/103 (0.0%)	94/4,731 (2.0%)	N/A		N/A		N/A		N/A	
Autoimmune thyroiditis	0/103 (0.0%)	42/4,731 (0.9%)	N/A		N/A		N/A		N/A	
**Low income (n = 3,105)**										

Hypothyroidism	4/93 (4.3%)	118/3,012 (3.9%)	0.91 (0.33-2.51)	0.851	1.76 (0.59-5.32)	0.313	0.78 (0.24-2.53)	0.676	0.78 (0.24-2.52)	0.673
Hyperthyroidism	1/93 (1.1%)	53/3,012 (1.8%)	1.65 (0.23-12.05)	0.623	1.68 (0.22-12.63)	0.617	1.22 (0.16-9.53)	0.853	N/A	
Grave's disease	0/93 (0.0%)	5/3,012 (0.2%)	N/A		N/A		N/A		N/A	
Thyroiditis	0/93 (0.0%)	40/3,012 (1.3%)	N/A		N/A		N/A		N/A	
Autoimmune thyroiditis	0/93 (0.0%)	19/3,012 (0.6%)	N/A		N/A		N/A		N/A	
**Middle income (n = 2,347)**										
Hypothyroidism	1/59 (1.7%)	80/2,288 (3.5%)	2.10 (0.29-15.36)	0.464	3.76 (0.49-28.81)	0.202	0.28 (0.03-2.50)	0.257	0.30 (0.04-2.56)	0.271
Hyperthyroidism	1/59 (1.7%)	39/2,288 (1.7%)	1.01 (0.14-7.45)	0.996	1.17 (0.15-9.02)	0.879	1.57 (0.07-34.02)	0.773	N/A	
Grave's disease	0/59 (0.0%)	10/2,288 (0.4%)	N/A		N/A		N/A		N/A	
Thyroiditis	0/59 (0.0%)	35/2,288 (1.5%)	N/A		N/A		N/A		N/A	
Autoimmune thyroiditis	0/59 (0.0%)	18/2,288 (0.8%)	N/A		N/A		N/A		N/A	
**High income (n = 2,618)**										
Hypothyroidism	1/85 (1.2%)	80/2,533 (3.2%)	2.74 (0.38-19.87)	0.320	5.10 (0.60-43.50)	0.137	0.38 (0.05-3.28)	0.382	0.37 (0.04-3.13)	0.358
Hyperthyroidism	2/85 (2.4%)	38/2,533 (1.5%)	0.63 (0.15-2.66)	0.532	0.58 (0.13-2.63)	0.479	5.25 (1.07-25.87)	0.041	N/A	
Grave's disease	1/85 (1.2%)	4/2,533 (0.2%)	0.13 (0.02-1.20)	0.072	0.06 (0.01-0.68)	0.023	N/A		N/A	
Thyroiditis	0/85 (0.0%)	33/2,533 (1.3%)	N/A		N/A		N/A		N/A	
Autoimmune thyroiditis	0/85 (0.0%)	15/2,533 (0.6%)	N/A		N/A		N/A		N/A	
**CCI scores = 0 (n = 6,518)**										
Hypothyroidism	4/59 (6.8%)	200/6,174 (3.2%)	0.48 (0.17-1.33)	0.158	0.49 (0.16-1.53)	0.220	2.14 (0.66-6.94)	0.204	2.23 (0.70-7.12)	0.174
Hyperthyroidism	2/59 (3.4%)	101/6,174 (1.6%)	0.49 (0.12-2.04)	0.329	0.32 (0.07-1.44)	0.138	3.43 (0.72-16.25)	0.121	N/A	
Grave's disease	1/59 (1.7%)	15/6,174 (0.2%)	0.15 (0.02-1.13)	0.065	0.07 (0.01-0.70)	0.024	N/A		16.36 (1.39-192.44)	0.026
Thyroiditis	0/59 (0.0%)	85/6,174 (1.4%)	N/A		N/A		N/A		N/A	
Autoimmune thyroiditis	0/59 (0.0%)	40/6,174 (0.6%)	N/A		N/A		N/A		N/A	
**CCI scores = 1 (n = 889)**										
Hypothyroidism	0/56 (0.0%)	42/1,054 (4.0%)	N/A		N/A		N/A		N/A	
Hyperthyroidism	0/56 (0.0%)	17/1,054 (1.6%)	N/A		N/A		N/A		N/A	
Grave's disease	0/56 (0.0%)	2/1,054 (0.2%)	N/A		N/A		N/A		N/A	
Thyroiditis	0/56 (0.0%)	15/1,054 (1.4%)	N/A		N/A		N/A		N/A	

Autoimmune thyroiditis	0/56 (0.0%)	7/1,054 (0.7%)	N/A		N/A		N/A		N/A	
**CCI scores ≥ 2 (n = 663)**										
Hypothyroidism	2/122 (1.6%)	36/605 (6.0%)	3.76 (0.89-15.85)	0.071	3.42 (0.77-15.23)	0.107	0.32 (0.07-1.44)	0.138	0.31 (0.07-1.41)	0.131
Hyperthyroidism	2/122 (1.6%)	12/605 (2.0%)	1.20 (0.26-5.43)	0.815	0.49 (0.09-2.84)	0.430	2.38 (0.38-14.98)	0.356	N/A	
Grave's disease	0/122 (0.0%)	2/605 (0.3%)	N/A		N/A		N/A		N/A	
Thyroiditis	0/122 (0.0%)	8/605 (1.3%)	N/A		N/A		N/A		N/A	
Autoimmune thyroiditis	0/122 (0.0%)	5/605 (0.8%)	N/A		N/A		N/A		N/A	
**Non-hypertension (n = 6,413)**										
Hypothyroidism	2/77 (2.6%)	181/6,336 (2.9%)	1.10 (0.27-4.53)	0.892	1.15 (0.27-4.89)	0.855	1.70 (0.38-7.67)	0.489	1.48 (0.33-6.55)	0.610
Hyperthyroidism	0/77 (0.0%)	96/6,336 (1.5%)	N/A		N/A		N/A		N/A	
Grave's disease	0/77 (0.0%)	15/6,336 (0.2%)	N/A		N/A		N/A		N/A	
Thyroiditis	0/77 (0.0%)	76/6,336 (1.2%)	N/A		N/A		N/A		N/A	
Autoimmune thyroiditis	0/77 (0.0%)	34/6,336 (0.5%)	N/A		N/A		N/A		N/A	
**Hypertension (n = 1,657)**										
Hypothyroidism	4/160 (2.5%)	97/1,497 (6.5%)	2.70 (0.98-7.44)	0.055	3.21 (1.13-9.11)	0.029	0.40 (0.13-1.21)	0.105	0.39 (0.13-1.17)	0.093
Hyperthyroidism	4/160 (2.5%)	34/1,497 (2.3%)	0.91 (0.32-2.59)	0.854	0.74 (0.26-2.14)	0.577	3.52 (1.05-11.77)	0.041	N/A	
Grave's disease	1/160 (0.6%)	4/1,497 (0.3%)	0.43 (0.05-3.83)	0.446	0.34 (0.04-3.12)	0.339	N/A		18.67 (1.51-230.59)	0.023
Thyroiditis	0/160 (0.0%)	32/1,497 (2.1%)	N/A		N/A		N/A		N/A	
Autoimmune thyroiditis	0/160 (0.0%)	18/1,497 (1.2%)	N/A		N/A		N/A		N/A	

Abbreviations: CCI, Charlson comorbidity index; N/A, Not applicable

* Unconditional logistic regression model, Significance at P < 0.05

† Model 1 was adjusted for age, sex, income, CCI scores and hypertension.

‡ Model 2 was adjusted for model 1 plus hypothyroidism, hyperthyroidism, and thyroiditis.

§ Model 3 was adjusted for model 1 plus hypothyroidism, Grave's disease, and autoimmune thyroiditis.
